# Ablação por Campo Pulsado no Brasil: O que o Registro nos Diz e o que Ele Não Diz

**DOI:** 10.36660/abc.20260176

**Published:** 2026-05-18

**Authors:** Fatih Aydin

**Affiliations:** 1 TC Sağlik Bakanliği Eskişehir Şehir Hastanesi Odunpazari Turquia TC Sağlik Bakanliği Eskişehir Şehir Hastanesi, Odunpazari – Turquia

**Keywords:** Fibrilação Atrial, Brasil

O artigo Ablação por Campo Pulsado para Fibrilação Atrial: Experiência Multicêntrica Inicial no Brasil, publicado recentemente nesta revista por May et al., é uma contribuição oportuna e verdadeiramente importante.^1^ Documentar os 394 procedimentos iniciais de ablação por campo pulsado (ACP) em 60 centros e 60 operadores não é tarefa fácil, e os autores merecem reconhecimento por organizar um esforço voluntário e multicêntrico tão cedo na adoção da tecnologia no país. A ausência de complicações graves e a demonstração de uma curva de aprendizado rápida e acentuada são achados tranquilizadores para centros que ainda consideram a adoção da ACP.

Dito isso, alguns aspectos do registro merecem uma discussão mais aprofundada.

Em primeiro lugar, a natureza voluntária da submissão de dados é uma limitação significativa e, talvez, mais consequente do que os autores reconhecem. Registros baseados em autorrelato são inerentemente suscetíveis a viés de seleção: operadores que encontram complicações ou resultados subótimos podem simplesmente não submeter os dados. Os autores observam essa possibilidade, mas a ausência de qualquer mecanismo para quantificá-la significa que a taxa de complicações de zero deve ser interpretada com a devida cautela. O registro MANIFEST-PF, que relatou uma taxa de complicações graves de 2,1% em uma coorte europeia muito maior, nos lembra que nenhuma técnica de ablação está totalmente isenta de riscos.^2^

Em segundo lugar, se destaca o uso quase universal de anestesia geral (98%). Na Europa, a sedação profunda é a norma para a ACP na maioria dos centros, e a experiência do estudo MANIFEST-PF sugere que ela não compromete os resultados.^2^ No Brasil, a preferência pela anestesia geral pode refletir a disponibilidade de suporte anestésico em centros urbanos de grande volume, mas também levanta uma preocupação prática. Se a ACP pretende cumprir sua promessa de melhorar o acesso à ablação por cateter em um país onde 5 milhões de pessoas vivem com fibrilação atrial,^3^ a escalabilidade desse modelo anestésico precisa ser analisada criteriosamente. Os centros fora dos grandes centros urbanos, que são justamente os mais carentes, podem não ter a infraestrutura necessária para replicá-lo.

Em terceiro lugar, a taxa de 26% de lesões adicionais necessárias após o remapeamento, predominantemente em centros de menor volume, merece mais do que uma breve menção. Esse número tem implicações diretas para o sucesso do procedimento e, em última análise, para a recorrência de arritmias a longo prazo. O estudo ADVENT, conduzido em condições mais padronizadas, relatou 73,3% de ausência de um desfecho composto em um ano. Resta saber se a coorte brasileira alcançará taxas comparáveis. A orientação apenas por fluoroscopia, utilizada em 22% dos procedimentos, pode contribuir para o isolamento incompleto; os autores reconhecem essa preocupação, mas ela exige uma análise mais estruturada.^4,5^

Por fim, a análise da curva de aprendizado, embora interessante, foi derivada de apenas 125 dos 394 procedimentos devido à falta de dados. Isso reduz a confiança nas estimativas de platô de 10 a 15 procedimentos, que podem ser otimistas para operadores em centros de menor volume com perfis de pacientes mais complexos.

Nenhum desses pontos diminui o valor do registro. No entanto, eles ressaltam que os dados atuais representam um ponto de partida, não uma conclusão. O acompanhamento prospectivo com notificação obrigatória, protocolos de imagem padronizados e pelo menos 12 meses de monitoramento do ritmo cardíaco fortaleceriam substancialmente o que já é uma base promissora para a ACP no Brasil.
