# Supplementary material for: Ablação por Campo Pulsado no Brasil: O que o Registro nos Diz e o que Ele Não Diz
Source: Arq Bras Cardiol. 2026 May 18;123(5):e20260176. [Article in Portuguese] doi: 10.36660/abc.20260176 (PMC13399089; doi:10.36660/abc.20260176)
Supplement: Translation

# Translation: Pulsed Field Ablation in Brazil: What the Registry Tells Us, and What It Does Not

**DOI:** 10.36660/abc.20260176i

**Authors:** Fatih Aydin

**Affiliations:** 1 Odunpazari Türkiye TC Sağlik Bakanliği Eskişehir Şehir Hastanesi, Odunpazari – Türkiye

**Keywords:** Atrial Fibrillation, Brazil

The article Brazilian Registry of Pulsed Field Catheter Ablation for the Treatment of Patients with Atrial Fibrillation, recently published in this journal by May et al., is a timely and genuinely important contribution.^1^ Documenting the initial 394 pulsed field ablation (PFA) procedures across 60 centers and 60 operators is no small task, and the authors deserve credit for organizing a voluntary, multicenter effort this early in the technology's domestic adoption. The absence of major complications and the demonstration of a steep, short learning curve are reassuring findings for centers still considering whether to adopt PFA.

That said, a few aspects of the registry warrant further discussion.

First, the voluntary nature of data submission is a meaningful limitation, and perhaps a more consequential one than the authors acknowledge. Registries built on self-reporting are inherently susceptible to selection bias: operators encountering complications or suboptimal outcomes may simply not submit. The authors note this possibility, but the absence of any mechanism to quantify it means the complication rate of zero should be interpreted with appropriate caution. The MANIFEST-PF registry, which reported a 2.1% major complication rate across a far larger European cohort, reminds us that no ablation technique is entirely free of risk.^2^

Second, the near-universal use of general anesthesia (98%) stands out. In Europe, deep sedation is the norm for PFA in most centers, and the MANIFEST-PF experience suggests it does not compromise outcomes.^2^ In Brazil, the preference for general anesthesia may reflect the availability of anesthesiology support at high-volume urban centers, but it also raises a practical concern. If PFA is to fulfill its promise of improving access to catheter ablation in a country where 5 million people live with atrial fibrillation,^3^ the scalability of this anesthesia model needs scrutiny. Facilities outside major urban centers, which are precisely those most underserved, may lack the infrastructure to replicate it.

Third, the 26% rate of additional lesions required after remapping, predominantly in lower-volume centers, deserves more than a brief mention. This figure has direct implications for procedural success and, ultimately, for long-term arrhythmia recurrence. The ADVENT trial, conducted under more standardized conditions, reported a 73.3% one-year freedom from a composite endpoint. Whether the Brazilian cohort will approach comparable rates remains unknown. Fluoroscopy-only guidance, used in 22% of procedures, may be a contributor to incomplete isolation; the authors acknowledge this concern, but it calls for a more structured analysis.^4,5^

Finally, the learning curve analysis, while interesting, was derived from only 125 of the 394 procedures due to missing data. This reduces confidence in the plateau estimates of 10–15 procedures, which may be optimistic for operators at lower-volume centers with more complex patient profiles.

None of these points diminishes the registry's value. But they do highlight that the current data represent a starting point, not a conclusion. A prospective follow-up with mandatory reporting, standardized imaging protocols, and at least 12 months of rhythm follow-up would substantially strengthen what is already a promising foundation for PFA in Brazil.
