# Supplementary material for: Ablação por Campo Pulsado no Brasil: O que o Registro nos Diz e o que Ele Não Diz
Source: Arq Bras Cardiol. 2026 May 18;123(5):e20260176. [Article in Portuguese] doi: 10.36660/abc.20260176 (PMC13399089; doi:10.36660/abc.20260176)
Supplement: Related texts

# Reply: Carta-resposta

**Authors:** Bruna Miers Miers May, Nilson Araujo, Jacob Atie, Silvia Helena Cardoso Boghossian, Angelo Amato Vincenzo De Paola, Andre d’Avila, Carina Abigail Hardy, Washington A. Maciel, José Tarcísio Medeiros de Vasconcelos, Claudio Munhoz, Alexander Romeno Janner Dal Forno, Eduardo B. Saad, Mauricio Scanavacca, Andre Zimerman

**Affiliations:** 1 Hospital SOS Cardio Florianópolis SC Brasil Hospital SOS Cardio, Florianópolis, SC – Brasil; 2 Rede d’Or São Luiz Hospitais Rio de Janeiro RJ Brasil Rede d’Or São Luiz Hospitais, Rio de Janeiro, RJ – Brasil; 3 Universidade Federal do Rio de Janeiro Hospital Universitário Clementino Fraga Filho Rio de Janeiro RJ Brasil Universidade Federal do Rio de Janeiro – Hospital Universitário Clementino Fraga Filho, Rio de Janeiro, RJ – Brasil; 4 Hospital Universitario Pedro Ernesto Rio de Janeiro RJ Brasil Hospital Universitario Pedro Ernesto, Rio de Janeiro, RJ – Brasil; 5 Hospital Vitoria Rio de Janeiro RJ Brasil Hospital Vitoria, Rio de Janeiro, RJ – Brasil; 6 Hospital São Paulo Escola Paulista de Medicina São Paulo SP Brasil Hospital São Paulo – Escola Paulista de Medicina, São Paulo, SP – Brasil; 7 Universidade de São Paulo Instituto do Coração do Hospital das Clínicas Faculdade de Medicina São Paulo SP Brasil Instituto do Coração do Hospital das Clínicas da Faculdade de Medicina da Universidade de São Paulo, São Paulo, SP – Brasil; 8 Clínica São Vicente Rio de Janeiro RJ Brasil Clínica São Vicente – Rede d’Or, Rio de Janeiro, RJ – Brasil; 9 Hospital Beneficência Portuguesa de São Paulo São Paulo SP Brasil Hospital Beneficência Portuguesa de São Paulo, São Paulo, SP – Brasil; 10 Hospital Samaritano Rio de Janeiro RJ Brasil Hospital Samaritano, Rio de Janeiro, RJ – Brasil; 11 Hospital Moinhos de Vento Porto Alegre RS Brasil Hospital Moinhos de Vento, Porto Alegre, RS – Brasil

Gostaríamos de agradecer a carta atenciosa e os comentários construtivos referentes ao nosso artigo.^1^ Concordamos fundamentalmente com as observações, que destacam com precisão áreas críticas para a evolução futura dessa tecnologia no Brasil.

Como os autores corretamente apontam, a natureza voluntária do nosso registro é uma limitação significativa que o torna inerentemente suscetível a viés de seleção. Operadores que encontrarem complicações podem simplesmente optar por não submeter seus dados. Portanto, concordamos plenamente que nossa taxa de complicações relatada de zero deve ser interpretada com a devida cautela. À medida que a adoção da ablação por campo pulsado (ACP) se expande por diversos centros no Brasil, prevemos que as taxas de complicações no mundo real provavelmente se alinharão mais de perto com coortes maiores, como o registro europeu MANIFEST-PF, que relatou uma taxa de complicações maiores de 2,1%. Isso serve como um lembrete vital de que nenhuma técnica de ablação é completamente isenta de riscos.^2^

Compartilhamos também da preocupação com o uso quase universal de anestesia geral, utilizada em 98% dos casos da nossa coorte. Embora a anestesia geral garanta a imobilidade do paciente e maximize a precisão durante a fase inicial de aprendizado de uma nova tecnologia, ela exige infraestrutura e pessoal especializados. Centros fora dos grandes centros urbanos podem não possuir a infraestrutura necessária para replicar essa abordagem, tornando a transição gradual para a sedação profunda, prática comum em muitos centros europeus, uma consideração importante para o futuro.

Além disso, a carta destaca com precisão a taxa de 26% de lesões adicionais necessárias após o remapeamento. Concordamos que esse número tem implicações diretas para o sucesso do procedimento e, em última análise, para a recorrência de arritmias a longo prazo. A dependência exclusiva da fluoroscopia, que ocorreu em 22% dos nossos procedimentos, pode de fato contribuir para o isolamento incompleto. A integração do mapeamento eletroanatômico e da ecocardiografia intracardíaca pode aprimorar a precisão da identificação das lesões, e o estabelecimento de protocolos de imagem padronizados será fundamental daqui para frente.

Em relação à análise da curva de aprendizado, os autores observaram corretamente que ela foi derivada de um subconjunto menor de procedimentos, precisamente 125 dos 394 casos totais. Gostaríamos de esclarecer a metodologia por trás disso: a análise da curva de aprendizado foi restrita a esse subconjunto menor de pacientes porque estabelecemos um requisito rigoroso de que um operador deveria ter realizado um mínimo de 20 casos para que sua curva de aprendizado fosse avaliada estatisticamente. Os procedimentos de operadores que ainda não haviam atingido esse limite foram naturalmente excluídos desta subanálise específica. No entanto, reconhecemos prontamente que o platô observado de 10 a 15 procedimentos pode ser otimista para operadores que lidam com perfis de pacientes mais complexos em ambientes de menor volume.

Em última análise, concordamos com a conclusão de que nossos dados atuais representam um ponto de partida, e não uma resposta definitiva. O estabelecimento de um registro prospectivo de acompanhamento com notificação obrigatória, protocolos de imagem padronizados e pelo menos 12 meses de acompanhamento do ritmo cardíaco é o próximo passo necessário para fortalecer substancialmente a base da ACP no Brasil. Agradecemos a oportunidade de esclarecer esses pontos e permanecemos comprometidos com o avanço do uso seguro e eficaz dessa tecnologia.

## References

[1] May BM, Araujo N, Boghossian SHC, Atie J, Maciel WA, Hardy CA (2026). Brazilian Registry of Pulsed Field Catheter Ablation for the Treatment of Patients with Atrial Fibrillation. Arq Bras Cardiol.

[2] Turagam MK, Neuzil P, Schmidt B, Reichlin T, Neven K, Metzner A (2023). Safety and Effectiveness of Pulsed Field Ablation to Treat Atrial Fibrillation: One-Year Outcomes from the MANIFEST-PF Registry. Circulation.

# Reply

**Authors:** Bruna Miers Miers May, Nilson Araujo, Jacob Atie, Silvia Helena Cardoso Boghossian, Angelo Amato Vincenzo De Paola, Andre d’Avila, Carina Abigail Hardy, Washington A. Maciel, José Tarcísio Medeiros de Vasconcelos, Claudio Munhoz, Alexander Romeno Janner Dal Forno, Eduardo B. Saad, Mauricio Scanavacca, Andre Zimerman

**Affiliations:** 1 Florianópolis SC Brazil Hospital SOS Cardio, Florianópolis, SC – Brazil; 2 Rio de Janeiro RJ Brazil Rede d’Or São Luiz Hospitais, Rio de Janeiro, RJ – Brazil; 3 Rio de Janeiro RJ Brazil Universidade Federal do Rio de Janeiro – Hospital Universitário Clementino Fraga Filho, Rio de Janeiro, RJ – Brazil; 4 Rio de Janeiro RJ Brazil Hospital Universitario Pedro Ernesto, Rio de Janeiro, RJ – Brazil; 5 Rio de Janeiro RJ Brazil Hospital Vitoria, Rio de Janeiro, RJ – Brazil; 6 São Paulo SP Brazil Hospital São Paulo – Escola Paulista de Medicina, São Paulo, SP – Brazil; 7 São Paulo SP Brazil Instituto do Coração do Hospital das Clínicas da Faculdade de Medicina da Universidade de São Paulo, São Paulo, SP – Brazil; 8 Rio de Janeiro RJ Brazil Clínica São Vicente – Rede d’Or, Rio de Janeiro, RJ – Brazil; 9 São Paulo SP Brazil Hospital Beneficência Portuguesa de São Paulo, São Paulo, SP – Brazil; 10 Rio de Janeiro RJ Brazil Hospital Samaritano, Rio de Janeiro, RJ – Brazil; 11 Porto Alegre RS Brazil Hospital Moinhos de Vento, Porto Alegre, RS – Brazil

We would like to thank you for the thoughtful letter and constructive comments regarding our recent article.^1^ We fundamentally agree with their observations, which accurately highlight critical areas for the future evolution of this technology in Brazil.

As the authors rightly point out, the voluntary nature of our registry is a meaningful limitation that makes it inherently susceptible to selection bias. Operators encountering complications might simply choose not to submit their data. Therefore, we entirely agree that our reported complication rate of zero should be interpreted with appropriate caution. As the adoption of pulsed field ablation (PFA) expands across diverse centers in Brazil, we anticipate that real-world complication rates will likely align more closely with larger cohorts like the European MANIFEST-PF registry, which reported a 2.1% major complication rate. This serves as a vital reminder that no ablation technique is completely free of risk.^2^

We also share the concern regarding the near-universal use of general anesthesia, which was utilized in 98% of our cohort. While general anesthesia ensures patient immobility and maximizes precision during the initial learning phase of a novel technology, it requires specialized infrastructure and personnel. Facilities outside major urban centers may lack the infrastructure to replicate this approach, making a gradual transition toward deep sedation, which is the norm in many European centers, an important consideration for the future.

Furthermore, the letter accurately highlights the 26% rate of additional lesions required after remapping. We agree that this figure has direct implications for procedural success and, ultimately, long-term arrhythmia recurrence. Relying solely on fluoroscopy guidance, which occurred in 22% of our procedures, may indeed contribute to incomplete isolation. The integration of electroanatomic mapping and intracardiac echocardiography can enhance lesion accuracy, and establishing standardized imaging protocols will be vital moving forward.

Regarding the learning curve analysis, the authors correctly observed that it was derived from a smaller subset of procedures, precisely 125 out of the 394 total cases. We wish to clarify the methodology behind this: the learning curve analysis was restricted to this smaller subset of patients because we established a strict requirement that an operator must have performed a minimum of 20 cases for their learning curve to be statistically evaluated. Procedures from operators who had not yet met this threshold were naturally excluded from this specific sub-analysis. However, we readily acknowledge that the observed plateau of 10 to 15 procedures may be optimistic for operators managing more complex patient profiles in lower-volume settings.

Ultimately, we also agree with the conclusion that our current data represent a starting point rather than a definitive answer. Establishing a prospective follow-up registry with mandatory reporting, standardized imaging protocols, and at least 12 months of rhythm follow-up is the necessary next step to substantially strengthen the foundation of PFA in Brazil. We appreciate the opportunity to clarify these points and remain committed to advancing the safe and effective use of this technology.
